# 185 What matters most for physical activity participation? A national Delphi study identifying the top ten priorities of adolescents with physical disability

**DOI:** 10.1093/eurpub/ckae114.122

**Published:** 2024-09-26

**Authors:** Karen Brady, Ronan Cleary, Eva O’Gorman, Suzanne McDonough, Claire Kerr, Elaine McConkey, Jennifer Ryan, Ailish Malone

**Affiliations:** School of Physiotherapy, RCSI University of Medicine and Health Sciences, Dublin, Ireland; Central Remedial Clinic, Clontarf, Dublin 3, Ireland; School of Physiotherapy, RCSI University of Medicine and Health Sciences, Dublin, Ireland; Central Remedial Clinic, Clontarf, Dublin 3, Ireland; School of Physiotherapy, RCSI University of Medicine and Health Sciences, Dublin, Ireland; School of Nursing and Midwifery,Queen’s University Belfast, University Road. Belfast, Northern Ireland; Central Remedial Clinic, Clontarf, Dublin 3, Ireland; School of Physiotherapy, RCSI University of Medicine and Health Sciences, Dublin, Ireland; School of Physiotherapy, RCSI University of Medicine and Health Sciences, Dublin, Ireland

## Abstract

**Background:**

Most young people with disability are insufficiently active and do not meet the recommended physical activity guidelines. This can have a significant impact on future health. Supporting engagement in physical activity is challenging during adolescence, when priorities shift towards social participation. Finding ways to support sustainable and meaningful physical activity participation among adolescents with physical disability remains a challenge.

**Purpose:**

To establish a consensus among adolescents with physical disability regarding their priorities for enhancing their physical activity participation. The priorities will help inform the design of future interventions for physical activity participation.

**Methods:**

We conducted a multi-round Delphi study involving adolescents (13-17 years) with physical disability. Survey Round 1 comprised of open-ended questions, inviting free text responses. Free text responses were analysed thematically, creating items categorised to the Family of Participation-Related Constructs framework. In Round 2, participants rated the perceived importance of these items using a five-point Likert scale. The top 10 priorities were constructed from the highest-ranked items.

**Results:**

One-hundred-sixteen participants (mean age 14.6 years, range 13-17 years; 66 boys; 58 with cerebral palsy; 43 wheelchair users) completed Round 1. One-hundred-eight items were included in Round 2. Fifty-eight items were rated either “important” or “really important” by 70% of participants. The top 10 priorities were rated as “important” or “really important” by 82-94% of participants with a mean Likert score of 4.40 (range: 4.25-4.63). Seven of the top 10 related to the environmental context of the Family of Participation Related Constructs framework. The remaining three related to involvement and the related concept of preferences.

**Conclusion:**

The priorities identified will help inform the design and development of future physical activity interventions to support physical activity participation for adolescents with physical disability.

**Support/Funding Source:**

This work was funded by Health Research Board Ireland (HRCI-JFS-2022-006) via the Health Research Charities Ireland/Health Research Board Joint Funding Scheme 2022.

